# The predicament of the child refugee – understanding health and wellbeing in the daily life of migrant children and young people

**DOI:** 10.1080/17482631.2020.1843268

**Published:** 2020-12-09

**Authors:** Helena Korp, Live Stretmo

**Affiliations:** a Department of Social and Behavioral Sciences, University West, Trollhättan, Sweden; b Department of Education, Communication and Learning (IPKL), University of Gothenburg, Göteborg, Sweden

In this special issue of the International Journal of Qualitative Studies on Health and Well-being (QHW) we have invited scholars from different contexts and parts of the world to contribute their qualitative research undertakings concerning different aspects of health and wellbeing in regards to refugee children and young people.

According to the UNHCR a total of 70.6 million people are of “concern” globally, either as constituting what by the UNHCR is defined as *refugees according to the 1951 Geneva convention* (i.e., people forcibly displaced due to wars, violent internal conflicts and political persecution) or as *asylum-seekers, internally displaced people, stateless persons*, “returnees” and “others of concern”; meaning “individuals (…) to whom UNHCR extends its protection and/or assistance services, based on humanitarian or other special grounds” (UNHCR, 2019).

During year 2019 a total of 25.9 million people could be defined as Geneva refugees; over half of whom are under the age of 18 (UNHCR, 2019). Many of these children and young people have been forced to flee without their parents or close relatives; or have lost trace of them during the escape. They are so-called unaccompanied minors, for whom states have a specific set of obligations in-order to keep them safe from harm.

Other groups of refugee children and young people have never lived in what by host countries often is considered as a “country of origin”; an experience shared by many children and youngsters whose parents might have originated from for instance Afghan provinces and who have lived as irregulars in Iran or Pakistan during their childhoods; by children and young people growing up in refugee camps in neighbouring countries and by irregular migrant children with Latino backgrounds situated in the US.

The number of children possibly veiled by the other UNHCR categories of stateless people, asylum-seekers, “undocumented,” “returnees” and “others of concern” are more difficult to extract. Host-countries do not necessarily keep track or collect separable data on the number of possible children within these disparate categories.

In regards to a growing number of refugees during the last decade it is important to bring to mind that the vast majority of displaced people still live within their home countries or in neighbouring LGDP countries, with an irregular status and under extremely tough and unsafe circumstances. Devoid of care and of protection, the group constituting so-called unaccompanied children are often highlighted as particularly vulnerable, but it is also important to bring to mind that the perceived safety of children migrating together with parents or adult carers can be quite illusionary, especially for those with precarious legal status (See for instance, Andersson et al., 2010).

During the years following what from a European host-state of view has been highlighted as the “refugee crisis” (year of 2015), countries such as for instance Sweden have come to toughen their laws, policies and regulations in regards to the possibility of asylum-seekers to obtain a permanent residence permit. The negative impact of temporary stay and prolonged asylum processes on children and young people have been studied, but the knowledge seem short lived in relation to a regime of a toughening asylum climate.

This special issue highlights new research that examines the predicament of the child refugee from different angles, not least from that of the children themselves.

In this introduction, we intend to do a quick review of previous research concerning children and young people categorized as refugees. We have decided to analyze and define the concept of the “refugee” in a broad sense, not merely restricting us to a focus on the children categorized as refugees by a host-country. We also include studies focusing on children and young people in the midst of an asylum process, children for whom their legal status is not yet clear, undocumented children and so forth. Being categorized as either an asylum-seeker, or as a refugee or as an undocumented migrant, come to have a huge impact on the living conditions and wellbeing of a child. The different statuses associated with these disparate categories offer the individual either possible protection and citizen rights, or a life in exposure under the overarching risk of deportation. A one-eyed focus on children and young people who have obtained a refugee status in accordance with the principles stated in the Geneva convention would obscure the possible experiences of children and young people during their flight, their time spent as asylum-seekers, the life of undocumented children etc. Such a limited angle of incidence also risks obscuring how the way host-states decides to label or categorize some migrant children as refugees, whilst denying others this particular status, is always an act of politics.

In our introductory overview, we hence intend to look at literature covering the vast experiences of different children in various situations connected to the status or predicament of being a refugee, yet trying to avoid the risk of lumping together children and young people living under very different conditions.

The vast majority of research can be divided into four main fields of research (Cf. Derluyn & Broekaert, 2007; Watters, 2008; Wernesjö, 2011):

1. Interdisciplinary research focusing on *why* children migrate, their flight experiences and flight routs and different aspects of the specific national reception system in a given host-country;

Though sometimes criticized as being too macro-oriented, simplistic and deterministic (see for instance de Haas, 2011; Sassen, 2020; Skeldon, 1990), classical “push-pull” theories offer explanatory strength in order for scholars to answer the question why people, including children and youngsters, decide to migrate or flee and where they decide to go. According to Castles (2012), a major cause of today’s migration is the growing inequality in incomes between the world’s richest and poorest countries, paired with the level of human security that people find in their country of origin, i.e., freedom of thought and safety experienced by people living in so-called more- or less-developed countries. Other driving factors include uneven economic development, rapid demographic transitions, and technological advances in transport and communications, facilitating the moment of people between countries far apart.

From a macro-point of view there is probably no essential difference between the migration of adult refugees and that of children and young people.

A study conducted by Van Hear et al. (2020), drawing on the specific experiences of Afghan and Somali migrants’ movements, concludes that more mediating drivers, rather than merely looking studying mediating drivers in addtion to structural dimensions, appear to offer a greater potential in analysing why and were people choose to migrate. The study illustrates how deep-rooted cultural understandings and practices of migration reflect longstanding structural and economic inequalities between places of origin and places of destination. Hence conflict and insecurity make migration more likely, adding other drivers to the predisposing ones.

Some driving factors are still highlighted as more specific to children and young people; such as fleeing in order to escape violence at home, escaping a destiny as a child soldier, avoiding genital mutilation, forced marriage, etc. (See also Ayotte, 2000; Backlund, Eriksson, von Greiff & Åkerlund, 2012; Eide, 2000, 2005, ; Kohli, 2005; Kohli & Mitchell, 2007; Stretmo & Melander, 2013; Watters, 2008).

In both the cases of migration routes from Afghanistan and Somalia, international policy efforts have intended to mediate migration by inhibiting it. Instead of restricting migration, these actions have had the (unintended) consequence of rather forcing migrants to use irregular and clandestine channels (Van Hear et al., 2020; Watters, 2008,O’Connell Davidson, 2011), hence making the choice to flee an even more dangerous and hazardous undertaking.

(2) Paediatric or psychological research emphasizing the psychological wellbeing of refugee children in a new host country;

Research conducted within the medical and/or psychological field often highlights how many refugee children—regardless of being companied or unaccompanied by adult carers—suffer from traumas caused by events pre- and during their migration. Children may have witnessed violence and death closely, been subjected to violence themselves, or lost relatives or family members.

This more psychologically oriented research body, sometimes especially emphasizing unaccompanied children, has some-times been criticized for providing too little insight to and redundant of the daily lives of refugee children prior to their migration, but also of their everyday life strategies in the novel host country. Kohli (2005) and Kohli & Mitchell (2007) speculate if such a perception risks constructing and reproducing a negative stereotype of refugee children as vulnerable subjects—or victims—instead of active interpreters of their everyday life situations and contexts. There is hence a risk that refugee children are “othered” or constructed as essentially “different children” (Kohli 2007; Kohli & Mitchell, 2007. Cf. O’Connell Davidson, 2011). This also corresponds to what for instance, Engebrigtsen (2002, 2012) argues is a White Western view, where refugee children endanger being positioned on the outside of an implicit childhood concept, as some of the children and youngsters’ possible experiences—for instance, being separated at an early age, working instead of playing or going to school, experiencing rather traumatic ordeals—are considered as opposing the very notion of children and healthy or *normal* childhoods.

Wernesjö (2011: 504 f) further argues that a too one-sided focus on the possible emotional problems and vulnerability of refugee minors also risks to render invisible the structural conditions under which children and young people find themselves. The impact of asylum regulations, but also xenophobia, racism and/or social exclusion in everyday life, give different children and youngsters rather diverse possibilities in order to be included, integrated and continue their lives in a new country (Cf Fangen et al., 2020).

(3) A more legally oriented framework concentrating on the enforcement

of the rights of the child amid national policy and practice (see e.g., Connolly, 2011).

(4) Last a fourth and growing field of research emphasize the narratives of refugee children themselves; often connected to aspects of inclusion/exclusion, belonging and identity amongst refugee children reorienting themselves in a novel context.

This rather novel and growing research body shifts its main focus of incidence from the more macro-oriented explanations or the “psychologizing” accounts, to the micro context where refugee children and young people get by in their daily lives. This research also seeks to address questions regarding children and youngster’s conditions prior to their flight.

Many studies also emphasize how refugee children per see do not necessarily shift their social existence from one society to another, but rather seek to address just how children and young people maintain important and rather close transnational connections (see e.g., Melander & Shmulyar Gréen, 2018). Concepts such as “Skype motherhood” or “Skype fatherhood” can be helpful in order to understand the dynamics of how for instance, unaccompanied minors keep in contact with parents or carers situated abroad and how parents or carers continue being important in their children’s lives despite long distances.

According to Pinson et al. (2010), refugee children talk about experiences of “exclusion within (the arenas of) inclusion” such as being bullied in school and having trouble making new friends. These studies point to the everyday life experiences of unaccompanied minors and a striving to belong (Cf. Yuval-Davis, 2011) in a new context. Though “the longing to belong and the feeling that you belong somewhere are important emotional dimensions” (Wernesjö, 2011, p. 40), “belonginess” as such is not *a priori* given: the unaccompanied, refugee and asylum-seeking children and youngsters’ claim to belong endangers being challenged by others ascribing them a subordinated or even stigmatized positioning (Ibid; Pinson et al., 2010. Cf. Alinia, 2004; Yuval-Davis, 2011).

Other scholars highlight how post-migration stressors might in themselves endanger the wellbeing of refugee children; such as experiences of loneliness, loss and grief, and how for instance, extended judicial processes or the fear of or rejection to an asylum-application impacts on the wellbeing of children. Living as undocumented, experiencing interrupted schooling, a loss of social networks, the impact of parents’ mental distress, structural discrimination, struggling to achieve linguistic appropriation, experiences of prejudice and racism in the host-country etc. have further negative implications. While much has been done in many host-countries during the last five years in the health care and educational sectors to secure the human rights of refugee children, national and super-national refugee policy increasingly work against these rights (Cf. Svensson, 2017; Watters, 2008).

What this quick analysis of previous research put to the fore is the overwhelming lack of studies piloted from the LGDP-angle of incidence. The majority of studies on refugee-, conducted migrants- or asylum-seeking children are from a host-country perspective, i.e. from a context where the refugee child per see constitutes a double and ambiguous identity or a *double exposure*; as being both child and migrant (see e.g.,, Stretmo, 2014; Watters, 2008).

There is also a scarcity of research focusing the experiences of *companied refugee children* per see, as the predominant part of literature emphasizes the experiences of *unaccompanied minors* (Andersson et al., 2010; Stretmo, 2014). Another limitation is the lack of studies focusing on the children and young people who are rejected or deported from a host-country. What becomes of them?

There is also a seeming lack of studies taking the experiences of younger refugee children into account. The majority of studies tend to focus on older children and young people. This is of course partly due to the methodological difficulty of conducting research on small children, but is nevertheless an important unexplored area in a growing body of research.

This special edition contains nine articles covering different aspects of issues and studies related to the health and wellbeing of documented and undocumented refugee children and young people.

Whilst the majority of studies presented here are conducted in the north-western European context, the article written by Carmen Monico & David Duncan (2020) *Childhood narratives and the Lived Experiences of Hispanic and Latinx college students with uncertain immigration statuses in North Carolina*, is located in the USA. In order to analyze how insecurity during childhood years impacts the coping strategies and outlook on life of these young people, as well as their ability to gain and access to higher education, the authors interviewed 13 undocumented young adults. The majority of the young people interviewed in Monico and Duncan’s study are so-called DACAs (the Deferred Action for Childhood Arrivals programme), children that arrived the US as undocumented and were later granted a right to attend the K to 12 educational system in the US, despite their irregular status. Yet, during the last four years the criminalization or undocumented migrants in political rhetoric, pared with a hardening of migration regulations have come to constrain the rights of these DACAs, increasing the insecurity of their undocumented status. The article highlights the link between the experienced uncertainty of their immigration status during childhood and the health and well-being they experience as young adults.

In Brit Lynnebakke & Lutine de Wal Pastoor’s (2020) study *“It’s very hard, but I’ll manage.” Educational aspirations and educational resilience among recently resettled young refugees in Norwegian upper secondary schools*, the often rather optimistic and high educational aspirations articulated by young refugees, are the main focus. The findings suggest that there are various reasons (altruism, making their flight worth wile, personal growth etc.) behind their high levels of espoused motivation and aspirations, and that each individual could be motivated by a range of interrelated drivers. According to Lynnebakke and de Wal Pastoor, aspects of high aspirations need to be empirically and theoretically explored further. They further suggest that research into the educational aspirations of young immigrants should account for migration category and recognize that high educational aspirations as such is not a constant, but needs to be analyzed in regards to the different phases or stages of an asylum process.

Another of the contributions in this special issue focuses the language introduction (LI) program for newly arrived migrants aged 16–19 in Sweden. By asking how the introduction program is organized in order to enhance inclusion, Andreas Fejes & Magnus Dahlstedt (2020) paper *Language introduction as a space for the inclusion and exclusion of young asylum* *seekers in Sweden* draws on data collected from interviews conducted with newly arrived students, their teachers and principals at five different Swedish upper-secondary and so-called “Folk high schools.” Fejes & Dahlstedt’s study illustrates that inclusion versus exclusion is rarely a straight forward matter of either/or. Rather, processes of inclusion and exclusionary practices can coexist and include a multitude of dimensions, including rights and responsibilities, participation, and belonging.

Fatumo Osman, Abdikerim Mohamed, Georgina Warner & Anna Sarkadi (2020) in their paper *Longing for a sense of belonging—Somali immigrant adolescents’ experiences of their acculturation efforts in Sweden*, study aspects that can be analyzed as either supporting or hindering the inclusion of this large group. The study highlights how the participants express a “longing for a sense of belonging” in society and in school and emphasize several key considerations, such as a more holistic, inclusive approach to the individual needs

Osa Lundberg**’**s (2020^2020^) study *Defining and Implementing Social Integration: A case study of school leaders' and practitioners' work with newly arrived im/migrant and refugee* student and focus on the ideas of school leaders and –staff about social integration in relation to the education of newly arrived im/migrant and refugee students in Sweden. While education was often seen as a cruicial means for newly arrives pupils or students to achieve full integration, and students were constructed as vulnerable and exposed (due to for instance trauma, health issues or structural factors such as an insecure housing situation), the professionals in Lundberg’s study talked about their own role in regard to this student group exclusively in terms of “educational providers”. Focusing on academic outcomes, they constructed their role as disengaged with the possible psycho-social needs and well-being of the newly arrived students. Lundberg points to the risk that this stance puts the burden of social integration solely on the individual student, and fails to acknowledge the schools’ broader responsibilities for the academic as well as social inclusion and well-being, and the relatedness of these factors.

Hanna Ragnarsdottir's (2020) paper *Refugee families in Iceland: Opportunities and challenges in schools and society* shows that compared to other Nordic and European countries, Iceland forms a different context for resettlement, as immigration is very limited and well planned with ambitious policies for integration. Ragnarsdottir’s study is based on interviews with the parents who arrived as Syrian quota-refugees in 2016 with their children, and with teachers and principals in the children’s schools. She found that in spite of good intentions and high expectations on part of the society as well as the parents, children and families still faced a number of challenges that hinder full inclusion in the Icelandic society and school-system. These include schools’ inability to meet educational needs of children with interrupted schooling or trauma and to provide proper linguistic support as well as a lack of inter-ethnic peer-relations, but also parents’ insecurity about norms and values in the new society, loss of social networks and experiences of discrimination.

Barbara van der Ent, Jaco Dagevos & Talitha Stam (2020) in their pap*er Syrian-born children with a refugee background in Rotterdam. A child-centred approach to explore their social contacts and the experienced social climate in the Netherlands* explore different aspects of children’s resettlement in a Western host country after fleeing from Syria with their families, from the perspective of the children themselves. The authors used a board-game as a device to stimulate children (age 8–17) in focus-goups to share how they perceive their peer relations as well as the broader social climate in the host country. They found that while the participating children all reported that they had formed friendships in the new host-country, the majority of these were with peers who shared their ethnic background. In the context of the larger society, the children reported a dual experience of on the one hand feeling welcome, on the other hand excluded. Van der Ent and her co-authors found that the children were both involuntarily subjected to symbolic boundary drawing by others, and themselves engaged in boundary work.

Based on interviews with teachers and head masters, Mock-Muñoz de Luna, Granberg, Krasnik & Vitus (2020) in their paper *Towards more equitable education: Meeting health and wellbeing needs of newly arrived migrant and refugee children—perspectives from educators in Denmark and Sweden* look at how the health and wellbeing needs of children with refugee background are perceived and catered for in schools. Professionals in both contexts said on the one hand that they found that children with migration- and refugee background often have complex needs related to their migration-history. On the other hand, due to insufficient competency, resources and procedures, they found that the schools frequently fail to meet up to these needs. Whereas both policy and practice diverge between the two countries, Sweden having clearer standards and procedures and teachers expressing more confidence in their own ability to provide appropriate support, de Luna and her co-authors saw that the general lack of competencies, resources and procedures undermine educational equity as well as potentially reinforces migration-related health inequalities.

If children’s perspectives and experiences generally have been under- researched and under-represented in the body of literature on refugees and forced migration, some children are particularly invisible in the research as well as the society. Åsa Wahlström Smith's (2020) paper *Surviving through the kindness of strangers: Can there be ‘wellbeing’ among undocumented refugee children?* sheds light on the everyday-lives of children who live with “precarious status,” children who are part of families that alternate between seeking asylum and living as undocumented, and who live “hidden in plain sight” among us. The paper is based on ethnographic research with undocumented refugee children and their families in Sweden, and uses a range of methods including participant observations, interviews and children’s own photography. Wahlström Smith finds that these children’s legal and human rights and every aspect of their lives are conditioned by fear of exposure and deportability, as “the deportation regime” rather “the refugee protection regime” constitutes a social determinant of their health, negating children with precarious status the possibility of health and well-being in a real sense.
